# Purification, Characterization of Two Polysaccharides from *Pinelliae Rhizoma* Praeparatum Cum Alumine and Their Anti-Inflammatory Effects on Mucus Secretion of Airway Epithelium

**DOI:** 10.3390/ijms20143553

**Published:** 2019-07-20

**Authors:** Meibian Hu, Yujie Liu, Li Wang, Jiaolong Wang, Lin Li, Chunjie Wu

**Affiliations:** 1 School of Pharmacy, Chengdu University of Traditional Chinese Medicine, Chengdu 611137, China; 2 School of Pharmacy, Chengdu Medical College, Chengdu 610500, China

**Keywords:** *Pinelliae rhizoma* praeparatum cum alumine, polysaccharides, purification, characterization, mucus secretion

## Abstract

*Pinelliae Rhizoma* Praeparatum cum Alumine (PRPCA) is an important traditional processed herbal medicine mainly used for treating phlegm in China for more than 2000 years. In our previous studies, extraction optimization, characterization, and bioactivities of total polysaccharides from PRPCA were investigated. In this study, further purification of these polysaccharides was performed. Two polysaccharides named neutral fraction of total polysaccharides-II (TPN-II) and acidic fraction of total polysaccharides-II (TPA-II) were obtained by gradient ion-exchange chromatography followed by gel-permeation chromatography. Results of scanning electron microscopy (SEM) analysis in the present study showed that TPN-II had a tight structure with a rough and uneven surface, while TPA-II had a relative homogeneous surface and a loose structure. Further studies indicated that TPN-II was a homosaccharide mainly composed by glucose with a molecular weight of 8.0 kDa. TPA-II was mainly composed of mannose, rhamnose, glucuronic acid, galacturonic acid, glucose, galactose and arabinose in a molar ratio of 2.1, 2.3, 1.7, 10.6, 2.6, 14.2, and 2.5, with a molecular weight of 1250 kDa. The nuclear magnetic resonance (NMR) results indicated that α and β form glycoside bonds existed in TPN-II and TPA-II, and TPN-II was composed of α-glucopyranose. In addition, both purified polysaccharides have significant anti-inflammatory effects on mucus secretion of human airway epithelial NCI-H292 cells without cytotoxicity. Compared with TPN-II, TPA-II exhibited more significant anti-inflammatory effects on lipopolysaccharide (LPS)-induced airway inflammation by regulating levels of interleukin-4 (IL-4) and interferon-γ (IFN-γ) and inhibiting mucus secretion. The results suggest that polysaccharides from PRPCA could be explored as therapeutic agents in treating inflammation and over secretion of mucus in asthma.

## 1. Introduction

It is well known that polysaccharides are important biological macromolecules isolated from natural products [1]. In recent years, polysaccharides derived from natural plant medicines are considered as drug candidates for treating various diseases because they possess a wide spectrum of therapeutic properties, relatively low toxicities and minor side effects [2,3]. Many polysaccharides have been extensively studied, and reported to exhibit various biological activities, including immunomodulatory [4], antitumor [5], antioxidant [6], and anti-inflammatory [7] effects, etc.

The dried tuber of *Pinellia ternata* (Thunb.) Breit (Araceae), named Banxia in Chinese, is one of the famous traditional Chinese medicines (TCMs) [8]. It has been used for relieving cough and inflammation in China for more than 2000 years [9]. Modern pharmacological studies have revealed that Banxia possesses a variety of biological activities, such as anti-inflammatory, expectorant, antitussive, antitumor, antibacterial, and antioxidant effects, etc. [10,11]. Because of its toxicity, Banxia is traditionally used in clinic after processing. *Pinelliae Rhizoma* Praeparatum Cum Alumine (PRPCA) is one of the important traditional processed products of Banxia, mainly used for treating phlegm with lower toxicity than Banxia (The lethal dose for 50%(LD50) value of Banxia was 42.7 ± 1.27 g/kg, while LD50 value of PRPCA was more than 80 g/kg by intragastric administration in mice) [12,13]. According to the traditional processing technology, Banxia was processed with alumen as adjuvant material to obtain PRPCA [9,14].

**Figure 1:**
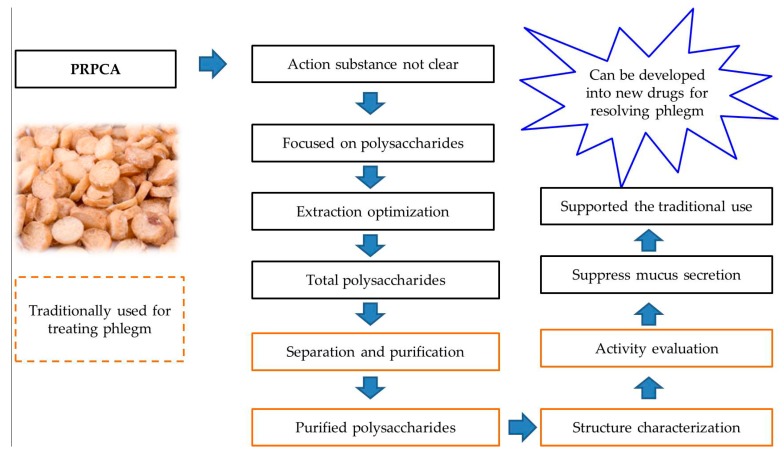
The research strategies of polysaccharides from *Pinelliae rhizoma* praeparatum cum alumine (PRPCA).

Although studies have proved the presence of lectins, alkaloids, flavonoids, fatty acids and phenylpropanoids in Banxia and PRPCA [12,15], the ingredients of their phlegm-resolving effects are still not completely clear. Our previous studies have found that PRPCA contains abundant polysaccharides with antioxidant and antimicrobial activities [14]. In this study, we focused on the purification, characterization and anti-inflammatory effects on mucus secretion of polysaccharides from PRPCA. A neutral polysaccharide (named TPN-II) and an acidic polysaccharide (named TPA-II) were obtained using gradient ion-exchange chromatography followed by gel-permeation chromatography. Their physicochemical properties were characterized by scanning electron microscopy (SEM), molecular weight determination, monosaccharide composition analysis, Fourier transform-infrared spectroscopy (FT-IR), and nuclear magnetic resonance (NMR) spectroscopy. Finally, we evaluated the in vitro anti-inflammatory effects of the two polysaccharides on mucus secretion in human airway epithelial cells (NCI-H292). The research strategies of polysaccharides from PRPCA were shown in Figure 1.

## 2. Results

### 2.1. Purification of Polysaccharides from PRPCA

**Figure 2:**
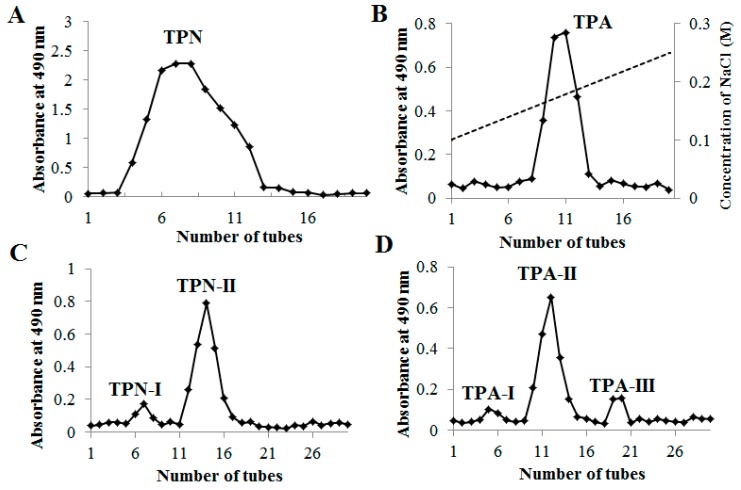
Elution curve of neutral fraction of total polysaccharides (TPN) (**A**) and acidic fraction of total polysaccharides (TPA) (**B**) on diethylaminoethyl cellulose 52 (DEAE-52) column, and elution curves of TPN-II (**C**) and TPA-II (**D**) on Sephadex-G50 and Sephacryl S-300 column, respectively.

The yield of polysaccharides from PRPCA was 13.21% using ultrasound-assisted extraction using optimum conditions based on the procedure reported in our previous study [14]. As shown in Figure 2, two fractions (TPN and TPA) were obtained by a diethylaminoethyl cellulose 52 (DEAE-52) column based on the different acidity. After further purification on Sephadex G-50 (TPN) and Sephacryl S-300 (TPA) columns with the deionized water as eluent, two purified polysaccharides of TPN-II and TPA-II were finally obtained.

### 2.2. Morphological Properties

**Figure 3:**
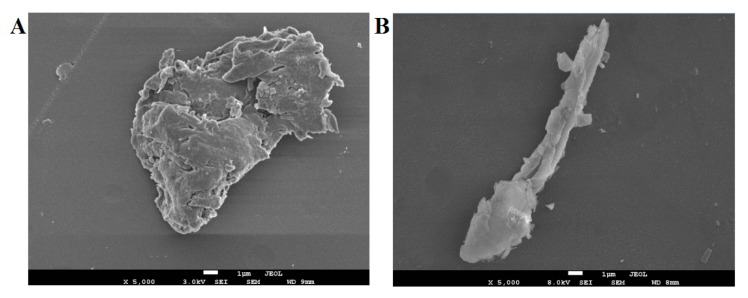
Scanning electron microscopy **(**SEM) images of neutral fraction of total polysaccharides-II (TPN-II) (**A**) and acidic fraction of total polysaccharides-II (TPA-II) (**B**). Morphology at 5000× (scalebar is 1 µm).

By observing the appearance, TPN-II was colorless and crystalline, while TPA-II had a light yellow color with larger viscosity than TPN-II. It’s reported that SEM was a qualitative tool to analyze the surface morphology of polysaccharides [16]. Therefore, the morphological properties of TPN-II and TPA-II were further observed by SEM in this study, and the results were shown in Figure 3. The surfaces of the two polysaccharides showed significant differences in size and shape when viewed by SEM. It showed that TPN-II had a tight structure with a rough and uneven surface. However, compared with TPN-II, TPA-II had a relative homogeneous surface and a loose structure. The differences in the morphological properties were according to the results of molecular weight of the polysaccharides, and may also be the reason for the difference in the activities [17].

### 2.3. The Characterization of TPN-II and TPA-II

#### 2.3.1. Ultraviolet (UV) Analysis

**Figure 4:**
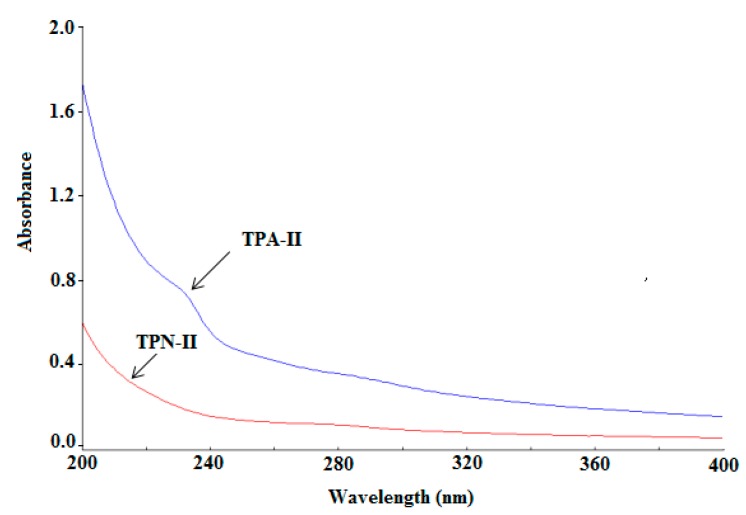
Ultraviolet (UV) spectrum of neutral fraction of total polysaccharides-II (TPN-II) and acidic fraction of total polysaccharides-II (TPA-II).

The results of ultraviolet (UV) spectrometric analysis were shown in Figure 4. There were no significant absorption peaks at 260 and 280 nm in the UV spectrum of TPA-II and TPN-II, indicating a low content of protein and nucleic acid impurity of the two purified polysaccharides.

#### 2.3.2. Molecular Weight and Monosaccharide Composition

**Figure 5:**
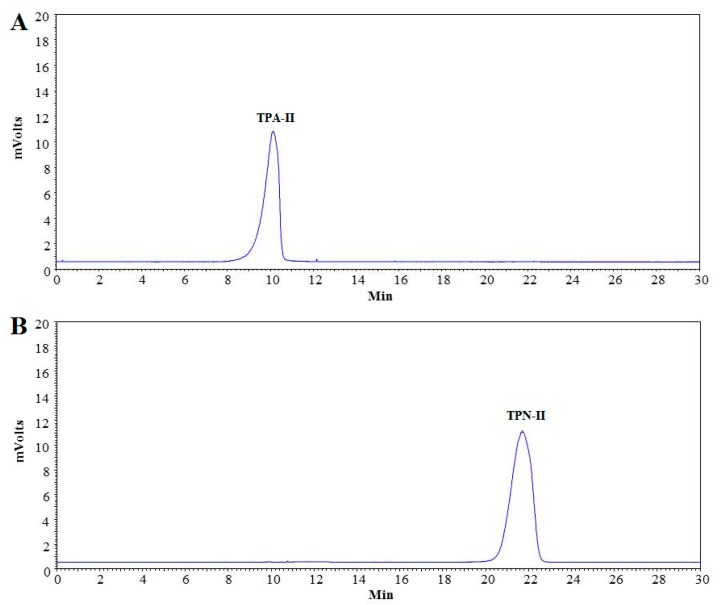
High performance gel permeation chromatography (HPGPC) profiles of neutral fraction of total polysaccharides-II (TPN-II) (**A**) and acidic fraction of total polysaccharides-II (TPA-II) (**B**).

The results of homogeneity of the purified polysaccharides were shown in Figure 5. From the HPGPC profiles of TPN-II and TPA-II, only a single symmetrical peak was detected, indicating that they are both homogeneous polysaccharides. By employing a calibration curve of Dextran standards (logM_W_ = −0.00313 X + 7.970, *R*^2^ = 0.999), the molecular weight of TPN-II and TPA-II was calculated to be 8.0 kDa and 1250 kDa, respectively.

**Figure 6:**
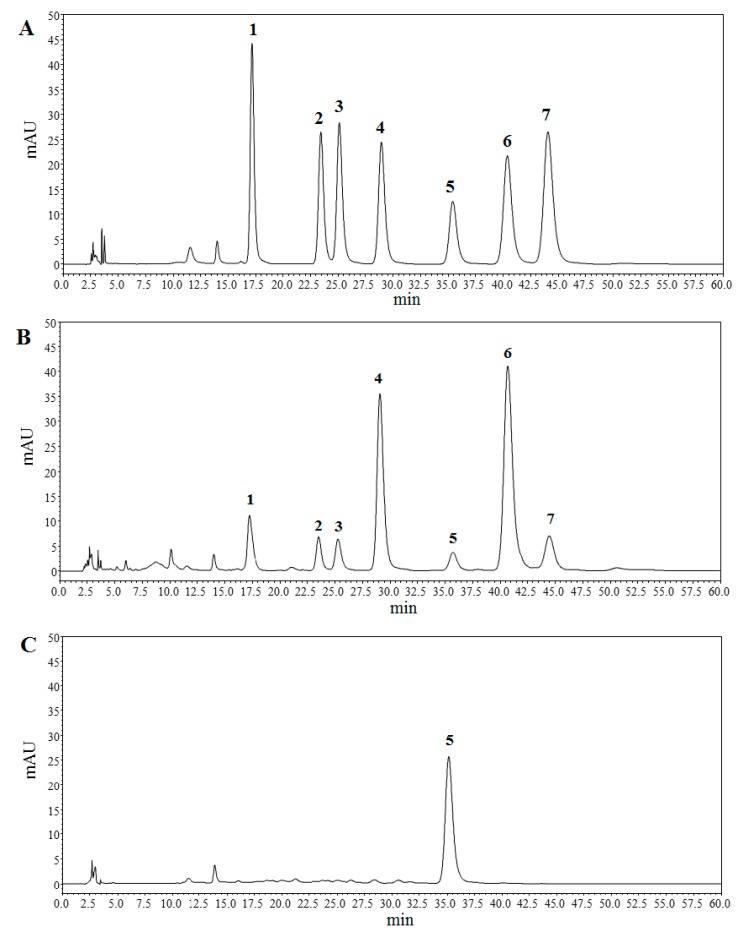
High performance liquid chromatography (HPLC) chromatograms of hydrolyzed polysaccharide derivatives. (**A**) is HPLC chromatogram of monosaccharide standards. (**B**) and (**C**) are HPLC chromatograms of acidic fraction of total polysaccharides-II (TPA-II) and neutral fraction of total polysaccharides-II (TPN-II), respectively. (1) mannose (Man), (2) rhamnose (Rha), (3) glucuronic acid (GluA), (4) galacturonic acid (GalA), (5) glucose (Glu), (6) galactose (Gal), (7) arabinose (Ara).

The monosaccharide compositions of TPN-II and TPA-II were determined by high performance liquid chromatography (HPLC), and the results were shown in Figure 6. The presences of the monosaccharides in hydrolyzed polysaccharide were identified by comparing with those of monosaccharide standards under the same conditions (Figure 6A). As a result, TPN-II was found to be mainly composed of glucose (Glu), indicating that it may be a homo-saccharide composed by glucose (Figure 6C). However, different from TPN-II, TPA-II was composed of mannose (Man), rhamnose (Rha), glucuronic acid (GluA), galacturonic acid (GalA), Glu, galactose (Gal) and arabinose (Ara) in a molar ratio of 2.1, 2.3, 1.7, 10.6, 2.6, 14.2, and 2.5 (Figure 6B).

To further clarify the structure of TPA-II and TPN-II, fourier transform-infrared spectroscopy (FT-IR) and NMR analysis of the two purified polysaccharides were performed (the results were shown in the Supplemental Materials). Based on the results of HPLC and NMR analysis, there were great differences in molecular weight, monosaccharide composition, branching degree, and content of uronic acid between TPN-II and TPA-II. Therefore, their biological activities may be varied due to the structural differences.

### 2.4. Anti-Inflammatory Effects on Mucus Secretion

#### 2.4.1. Effects of TPN-II and TPA-II on the Viability of NCI-H292 Cells

**Figure 7:**
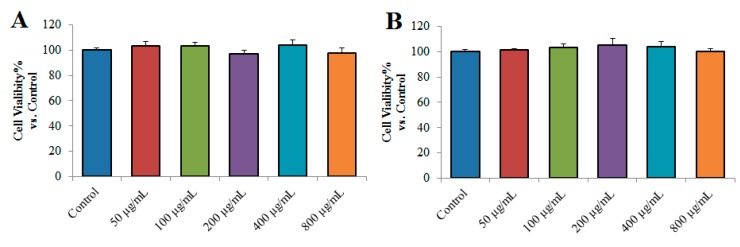
Effects of neutral fraction of total polysaccharides-II (TPN-II) (**A**) and acidic fraction of total polysaccharides -II (TPA-II) (**B**) on cell viability of human airway epithelial cells (NCI-H292).

The effects of TPN-II and TPA-II on the viability of NCI-H292 cells were studied first to investigate their toxicity. As shown in Figure 7, no significant effect of TPN-II and TPA-II was observed on the cell viability of NCI-H292 cells at the concentrations of 50 to 800 µg/mL. The results indicated that the two purified polysaccharides did not show any toxicity to NCI-H292 cells *in vitro* [18].

#### 2.4.2. Effects of TPN-II and TPA-II on Overproduction of MUC5AC, interleukin-4 (IL-4) and interferon-γ (IFN-γ) in NCI-H292 cells

**Figure 8:**
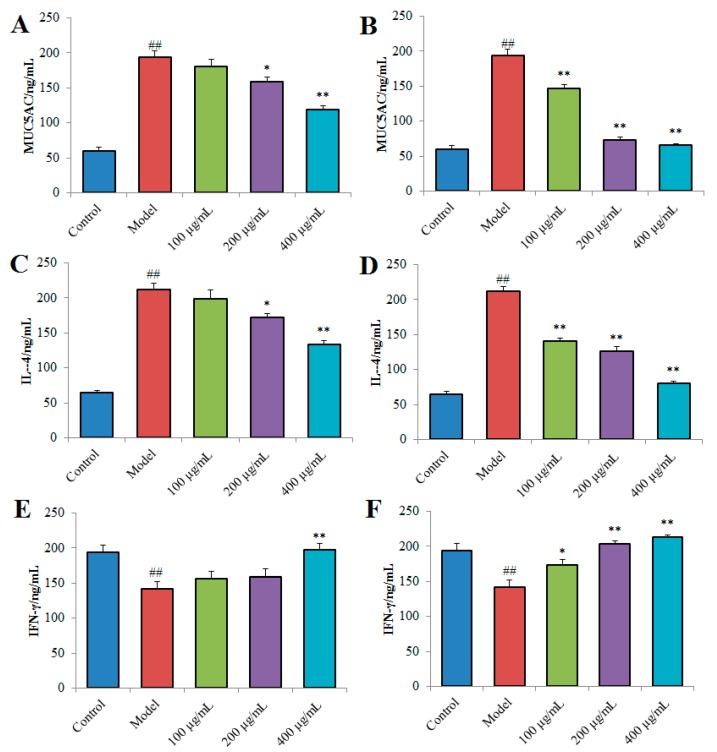
Effects of neutral fraction of total polysaccharides-II (TPN-II) (**A**), (**C**) and (**E**) and acidic fraction of total polysaccharides-II (TPA-II) (**B**), (**D**) and (**F**) on the levels of mucin-5 subtype AC (MUC5AC), interleukin-4 (IL-4) and Interferon-γ (IFN-γ) in the culture supernatants of human airway epithelial cells (NCI-H292). ## *p* < 0.01, compared with control group; * *p* < 0.05, ** *p* < 0.01, compared with model group.

In this study, mucin-5 subtype AC (MUC5AC) levels in the cellular secretions were evaluated by enzyme-linked immunosorbent assay (ELISA). As shown in Figure 8A,B, compared with cells in the control group, lipopolysaccharide (LPS)(1 µg/mL) treatment significantly induced the release of MUC5AC mucin in NCI-H292 cells. After treatment with TPN-II at the concentrations of 200 and 400 µg/mL, the MUC5AC mucin production was significantly decreased (*p* < 0.05 and *p* < 0.01). More importantly, TPA-II treatment at all tested concentrations suppressed the secretion of MUC5AC mucin (*p* < 0.01) with a concentration-dependent manner, compared with the cells stimulated by LPS in the model group.

The effects of TPN-II and TPA-II on the levels of interleukin-4 (IL-4) and interferon-γ (IFN-γ) in cell culture supernatants were presented in Figure 8C–F. Compared with the control group (without LPS treatment), we can see that LPS treatment sharply increased the level of IL-4 and decreased the level of IFN-γ in the model group (*p* < 0.01). Addition of different concentrations of TPN-II significantly decreased the excessive production of IL-4 (*p* < 0.05 and *p* < 0.01), while obviously increased the IFN-γ level at high concentration of 400 µg/mL (*p* < 0.01). Interestingly, TPA-II exhibited stronger effects on the levels of IL-4 and IFN-γ in the cell culture supernatants. The IL-4 content was significantly lower (*p* < 0.01), and IFN-γ content was significantly higher (*p* < 0.01) in all TPA-II treated group than in the model group.

#### 2.4.3. Effects of TPN-II and TPA-II on Expression of MUC5AC Protein

**Figure 9:**
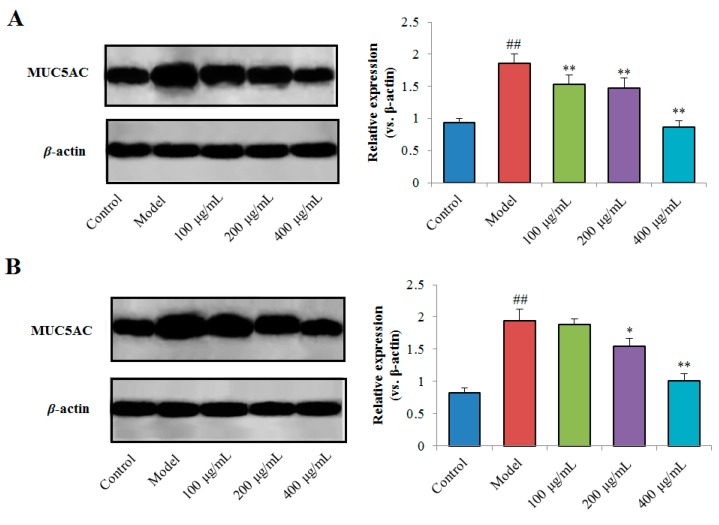
Effects of neutral fraction of total polysaccharides (TPN-II) (**A**) and acidic fraction of total polysaccharides (TPA-II) (**B**) on the expression of mucin-5 subtype AC (MUC5AC) protein in human airway epithelial cells (NCI-H292). ## *p* < 0.01, compared with control group; * *p* < 0.05, ** *p* < 0.01, compared with model group.

To further confirm the effects of the purified polysaccharides on the expression of MUC5AC protein in human airway epithelial NCI-H292 cells, total proteins of the cells were prepared and the western blot analysis was performed. The results are shown in Figure 9. The expression of MUC5AC protein was obviously upregulated (*p* < 0.01) in the cells stimulated by LPS, compared with the control cells without any treatment. By treatment with TPN-II (100, 200 and 400 µg/mL), the MUC5AC expression in the cells were significantly downregulated (*p* < 0.01), compared with the cells in the model group. Furthermore, TPA-II (200 and 400 µg/mL) also had an obvious inhibitory effect on the expression of MUC5AC protein in NCI-H292 cells (*p* < 0.05 and *p* < 0.01).

## 3. Discussion

Previous reports have revealed that polysaccharides from TCMs were important natural sources for drug development, mainly because they had diverse structure, unique monosaccharide compositions, and significant biological activities [19]. As a commonly used processed product of Banxia, PRPCA has been used for resolving phlegm in China for a long history [9,14]. To the best of our knowledge, this is the first study of purification, structural analysis, and activity evaluation of polysaccharides from PRPCA. This study may be helpful in the development of biological active polysaccharides with potential for the therapeutic use of resolving phlegm or to be used as ingredients in functional foods.

It has been reported that mucus layers of the airway can help to resist against outside parasites and particles, and they are involved in the mucosal response to airway inflammation [20]. Many investigations have shown that overproduction of mucus can lead to narrowing of the airway and deterioration of asthma [21,22]. The increased expression of mucin subfamily genes induced the increase of mucus production, and until now, more than 20 mucin subfamily genes have been found. Among them, MUC5AC is proved to be the major subfamily, and overproduction of airway mucins (especially MUC5AC or mucin-2 (MUC2)) is signature of human asthma and murine models of asthma [23]. Therefore, regulation of MUC5AC expression in airway epithelial cells is a target for asthma treatment. In this study, effects of the purified polysaccharides on the mucus secretion induced by LPS were evaluated in NCI-H292 cells. The results indicated that TPN-II and TPA-II significantly suppressed the mucus secretion and the expression in NCI-H292 cells, and TPA-II had a stronger effect. The purified polysaccharides, especially TPA-II, could be served as potential agents in treating airway inflammation with over-secretion of mucus.

Although the techniques for diagnosing and treating diseases have significantly improved, the treatment of allergic diseases is still very difficult in modern medicine [24,25]. As a complex chronic inflammatory airway disease, allergic asthma involves a variety of inflammation cell types and molecules, such as eosinophils, lymphocytes, chemokines and cytokines, etc. [26]. Previous research has shown that allergic asthma and inflammation are closely correlated with overproduction of IL-4 and decreased production of IFN-γ [27]. Compared with helper T 2 (Th2) cytokines [IL-1β, IL-4, IL-5, and tumor necrosis factor-α (TNF-α)], helper T 1 (Th1) cytokines (IFN-γ and IL-12) are associated to antagonism of immunoglobulin E (IgE) synthesis and Th2 cell responses to restrain the progress of asthma [28]. There is dynamic balance between immune responses of Th1 and Th2 cytokines in normal physiological condition. However, when this balance is broken, asthma and inflammation will occur [29,30]. Therefore, regulating the balance of Th1/Th2 cytokines is important therapeutic mechanism of allergic asthma. In this study, TPA-II exerted more significant effects than TPN-II on decreasing the IL-4 level and increasing the IFN-γ level in the he cell culture supernatants. The results indicated that TPA-II may be developed into therapeutic drugs for allergic asthma by mechanisms of regulating the balance of Th1/Th2 cytokines.

In this study, the TPN-II and TPA-II were found to possess significant in vitro anti-inflammatory effects on mucus secretion of NCI-H292 cells. Previous studies have shown that the structural characteristics of polysaccharides, such as molecular weight, monosaccharide composition, conformation, glycoside bond and branching degree, are closely related to their biological activities. [31]. The results in this study indicated that TPA-II had larger molecular weight of 1250 kD, more complex monosaccharide composition composed of Man, Rha, GluA, GalA, Glu, Gal and Ara in a molar ratio of 2.1, 2.3, 1.7, 10.6, 2.6, 14.2, and 2.5. According to the above literatures on structure activity relationship of polysaccharides, the presence of uronic acid group, and higher branching degree and high molecular weight may be the reason why TPA-II had more significant anti-inflammatory effects by regulating levels of IL-4 and IFN-γ and inhibiting mucus secretion. Previous reports have some similar findings. Zhang et al. [32] reported two functional polysaccharides (named polysaccharides of *Polygonum multiflorum* (WPMP)-1 and WPMP-2)) from *Polygonum multiflorum*, and the results indicated that the immunomodulatory activity of WPMP-2 was markedly better than that of WPMP-1. Further analysis of structure-activity relationship showed that the molecular weight, content of uronic acid, and degree of branching of WPMP-2 were all higher than those of WPMP-1, and these factors were suggested to be the positive characteristics for the better immunostimulatory activity. In addition, many other reports also showed that uronic acid content and molecular weight might influence the activity of the polysaccharides [18,33,34]. More studies on the structure–activity relationship of the two polysaccharides will be done in the future.

Collectively, two polysaccharides of TPN-II and TPA-II were obtained from PRPCA by gradient ion-exchange chromatography followed by gel-permeation chromatography. TPN-II was a homo-saccharide only composed by glucose with a molecular weight of 8.0 kDa. TPA-II was mainly composed of Man, Rha, GluA, GalA, Glu, Gal and Ara in a molar ratio of 2.1, 2.3, 1.7, 10.6, 2.6, 14.2, and 2.5, with a molecular weight of 1250 kDa. The NMR results indicated that α and β form glycoside bonds existed in TPN-II and TPA-II, and TPN-II was composed of α-glucopyranose. In addition, similar to most previously reported natural polysaccharides, TPN-II and TPA-II have significant anti-inflammatory effects on mucus secretion of human airway epithelial NCI-H292 cells without cytotoxicity. These results suggest that polysaccharides from PRPCA could be explored therapeutic agents in treating inflammation and over secretion of mucus in asthma.

## 4. Materials and Methods 

### 4.1. Materials and Reagents

PRPCA were purchased from Sichuan Neautus Traditional Chinese Medicine Co. Ltd. (Chengdu, China). A specimen was stored at School of Pharmacy, Chengdu University of Traditional Chinese Medicine (Chengdu, China). Cellulose DEAE-52 Sephadex G-50, and Sephacryl S-300 were purchased from GE healthcare (Fairfield, CT, USA). The monosaccharide standards of d-mannose (Man), d-glucose (Glu), l-galactose (Gal), l-rhamnose (Rha), d-galacturonic acid (GalA), d-glucuronic acid (GluA) and l-arabinose (Ara) were purchased from National Institute for Food and Drug Control (Beijing, China) 3-Methyl-1-phenyl-2-pyrazolin-5-one (PMP) was obtained from the SinoPharm Chemical Reagents Co., Ltd. (Shanghai, China). 3-(4,5-dimethylthiazol-2-yl)-2,5-diphenyltetrazolium bromide (MTT), dimethyl sulfoxide (DMSO) and lipopolysaccharide (LPS) were purchased from Sigma Chemical Co. (MO, USA). ELISA kits for MUC5AC, IL-4, IFN-γ and horseradish peroxidase-conjugated secondary antibody were purchased from Beijing Biosynthesis Biotechnology Co., Ltd. (Beijing, China) Primary antibody of MUC5AC was obtained from GeneTex (San Antonio, TX, USA). All other chemicals and reagents used in this study were of analytical grade.

### 4.2. Extraction and Purification of Polysaccharides from PRPCA

The extraction of polysaccharides from PRPCA was performed using ultrasound-assisted extraction (UAE) as our previous report [14]. The dried crude polysaccharides were re-dissolved to a concentration of 20 mg/mL in deionized water. After centrifugation (10000 rpm, 5 min), the supernatants (10 mL) was loaded onto a Cellulose DEAE-52 column (2.6 cm × 40 cm). The column was eluted with a stepwise gradient with 300 mL of distilled water followed by 0.05, 0.10, 0.25, 0.50, and 1.0 M NaCl solutions at a flow rate of 1 mL/min. The eluate (10 mL per tube) was collected and detected by using the phenol-sulfuric acid method for carbohydrate determination [35]. Two main fractions of TPA and TPN were ultimately found, collected and chosen for further purification. The TPN fraction was further purified by a Sephadex G-50 column (2 cm × 50 cm) using deionized water as eluent at a flow rate of 5 mL/min, and a neutral polysaccharide TPN-II (500 mg) was obtained. The TPA fraction was further eluted on a Sephacryl S-300 column (2 cm × 50 cm), and an acidic polysaccharide (TPA-II) was obtained (200 mg) finally.

### 4.3. Characterization Analysis of TPN-II and TPA-II

#### 4.3.1. SEM Analysis

The shape and surface characteristics of TPN-II and TPA-II were observed using a JSM-7001F scanning electron microscope system (JEOL, Tokyo, Japan). The polysaccharide samples were glued on specimen stubs by coating with a thin layer of platinum under high vacuum conditions at 8.0 kV acceleration voltage. Images were obtained under the magnification of 5000×.

#### 4.3.2. UV and FT-IR Spectrometric Analysis

Each polysaccharide of TPA-II and TPN-II was dissolved in deionized water, and UV spectra were recorded on a TU-1901 ultraviolet visible spectrophotometer (Beijing Purkinje General Instrument Co., Ltd., Beijing, China) in the 200–400 nm range. For the FT-IR analysis, each polysaccharide was thoroughly mixed with KBr powder and pressed into a 1-mm pellet. The FT-IR spectra were recorded using a TENSOR 37 FT-IR spectrophotometer (Bruker, Ettlingen, Germany) at the frequency range of 500–4000 cm^−1^ [36,37].

#### 4.3.3. Molecular Weight Determination and Monosaccharide Composition Analysis

Each polysaccharide of TPA-II and TPN-II was dissolved in deionized water (5 mg/mL). The purity and molecular weights of the polysaccharide was determined by high performance gel permeation chromatography (HPGPC) [38,39]. The chromatographic conditions were as follows: An Agilent 1260 HPLC system (Agilent, Santa Clara, CA, USA) equipped with an evaporative light scattering detector (ELSD). The sample separation was performed on a TSK-Gel G4000 SWXL (7.8 mm × 30 cm, 8 µm, Tosoh Corp, Tokyo, Japan) column, and eluted with deionized water at a flow rate of 0.6 mL/min. The injection volume was 20 µL and the column temperature was set at 35 °C. A series of dextran standards were used to calibrate the linear regression.

Monosaccharide composition of the purified polysaccharides was determined by PMP pre-column derivatization method [40,41]. Briefly, each polysaccharide of TPA-II and TPN-II (10 mg) was hydrolyzed by trifluoroacetic acid (TFA, 2 mol/L) at 110 °C for 5 h. Subsequently, the hydrolysate of the polysaccharide fractions were heated with PMP methanol solution (0.5 mol/L, 0.2 mL) and NaOH solution (0.3 mol/L, 0.2 mL) at 70 °C for 1 h. The mixture was extracted by trichloromethane (1 mL) for three times after being neutralized with HCl solution (0.3 mol/L, 0.2 mL). The aqueous phase was collected and diluted with deionized water to 5 mL. The samples were analyzed on an Agilent 1260 HPLC system equipped with an UV detector using a CAPCELL PAK MG II S5 C18 column (4.6 mm × 250 mm, 5 µm). The mobile phase was a mixture of acetonitrile (18:82, *v*/*v*) and phosphate buffer (0.05 mol/L, pH = 6.8) at a flow rate of 0.8 mL/min at 30 °C. The injection volume was 10 µL and wavelength was set at 245 nm.

#### 4.3.4. NMR Spectroscopy

TPN-II or TPA-II (30 mg) was dissolved in 0.5 mL deuteroxide (D_2_O) at room temperature for 3 h before NMR analysis. ^1^H and ^13^C NMR spectra were recorded on a Bruker Ascend^TM^ 600MHz spectrometer (Germany) at 30 °C.

### 4.4. Anti-Inflammatory Effects on Mucus Secretion of NCI-H292 Cells

#### 4.4.1. Cell Culture and Treatments

The human airway epithelial cell line NCI-H292 was obtained from the American Type Culture Collection (Manassas, VA, USA). Cells were cultured in RPMI-1640 culture medium (Sigma Aldrich, St. Louis, MO, USA) supplemented with 10% fetal bovine serum (FBS, Gibco-BRL Life Technologies, Grand Island, NY), 100 units/mL penicillin, and 100 µg/mL streptomycin in a 5% CO_2_ incubator at 37 °C. The adherent cells were subcultured every 3–4 days by treatment with trypsin-EDTA solution (Gibco-CRL).

Effects of TPA-II and TPN-II on cell viability of NCI-H292 cells were performed using MTT assay according to the previous report [42,43]. For further experiments of ELISA and western blot, cells (5 × 10^5^ cells/well) were seeded into six-well plates until confluent and then serum-starved for 24 h. After that, cells were treated with TPA-II or TPN-II (dissolved in RPMI-1640 culture medium) for 30 min, and then stimulated with 1 µg/mL LPS for 24 h. Untreated cells served as control, and cells treated with LPS alone served as model.

#### 4.4.2. ELISA for MUC5AC, IL-4 and IFN-γ

Cell culture supernatants were collected after incubation, and production of MUC5AC protein, IL-4 and IFN-γ in cell culture supernatants were measured by ELISA according to the manufacturer’s instructions.

#### 4.4.3. Western Blot Analysis

The cells were collected and total proteins were extracted by lysis buffer on ice. Protein content of samples was determined by the BCA Protein Assay Kit (Nanjing Jiancheng Bioengineering Institute, Nanjing, China). Western Blot Analysis was carried out as previously reported [44]. Briefly, equal amounts of protein (50 µg) were separated by 8% sodium dodecyl sulfate-polyacrylamide gel electrophoresis (SDS-PAGE) and transferred to polyvinylidene fluoride (PVDF) membranes (Millipore, Bedford, MA, USA). After blocking with 5% skimmed milk, the membranes were then incubated with the corresponding primary antibodies (1:1000 dilution) overnight at 4 °C, followed by horseradish peroxidase-conjugated secondary antibody for 1 h at room temperature. Protein bands were visualized using the enhanced chemiluminescence reagent (Beijing Biosynthesis Biotechnology, Beijing, China), and protein expression levels were normalized to that of β-actin.

### 4.5. Statistical Analysis

Data are presented as mean ± standard deviation (S.D.) (*n* = 3). Data were analyzed using Statistical Product and Service Solutions (SPSS) software version 17.0 (SPSS Inc., Chicago, IL, USA). Analysis of variance (ANOVA) was used to determine statistically significant differences between groups (*p* < 0.01)

## Supplementary Materials

Supplementary materials can be found at https://www.mdpi.com/1422-0067/20/14/3553/s1.

## Abbreviations

Ara	Arabinose
D2O	Deuteroxide
DEAE-52	Diethylaminoethyl Cellulose 52
ELISA	Enzyme-linked immunosorbent assay
Gal	Galactose
FT-IR	Fourier transform-infrared spectroscopy
GalA	Galacturonic acid
Glu	Glucose
GluA	Glucuronic acid
HPGPC	High performance gel permeation chromatography
HPLC	High performance liquid chromatography
IFN-γ	Interferon-γ
IgE	Immunoglobulin E
IL-4	Interleukin-4
LD50	Lethal dose for 50%
LPS	Lipopolysaccharide
Man	Mannose
MUC2	Mucin-2
MUC5AC	Mucin-5 subtype AC
NMR	Nuclear magnetic resonance
PRPCA	Pinelliae Rhizoma Praeparatum cum Alumine
PVDF	Polyvinylidene fluoride
Rha	Rhamnose
S.D.	Standard deviation
SDS-PAGE	Sodium dodecyl sulfate-polyacrylamide gel electrophoresis
SPSS	Statistical Product and Service Solutions
SEM	Scanning electron microscopy
Th1	Helper T 1
Th2	Helper T 2
TNF-α	Tumor necrosis factor-α
TPA	Acidic fraction of total polysaccharides
TPN	Neutral fraction of total polysaccharides
UV	Ultraviolet
WPMP	Polysaccharides of *Polygonum multiflorum*
