# Supplementary material for: Purification, Characterization of Two Polysaccharides from Pinelliae Rhizoma Praeparatum Cum Alumine and Their Anti-Inflammatory Effects on Mucus Secretion of Airway Epithelium
Source: Int J Mol Sci. 2019 Jul 20;20(14):3553. doi: 10.3390/ijms20143553 (PMC6678706; doi:10.3390/ijms20143553)
Supplement: Supplementary file 1 [file ijms-20-03553-s001.pdf]

Supplementary Materials

1. Results of FT-IR analysis

The FT-IR spectra of the two purified polysaccharides were shown in Figure 1. The results indicated that TPN-II (Figure 1A) and TPA-II (Figure 1B) possessed characteristic peaks of polysaccharides in the 400 – 4000 cm^{-1} region [1]. The broad and strong band around 3434 cm^{-1} represented the stretching vibrations of the –OH groups, and the band around 2925 cm^{-1} represented the C-H stretching and bending vibrations [2]. The absorption peaks at approximately 1747 cm^{-1} was related with the C=O stretching of ester bonds, indicating the presence of uronic acids in TPA-II [3]. The peak at approximately 1643 cm^{-1} was derived from C-O asymmetric stretching vibration [4]. In addition, the absorptions between 1000 and 1200 cm^{-1} were assigned to the stretching vibrations of the C-O-H side groups and the C-O-C glycosidic bond vibrations in the pyran structure [5].

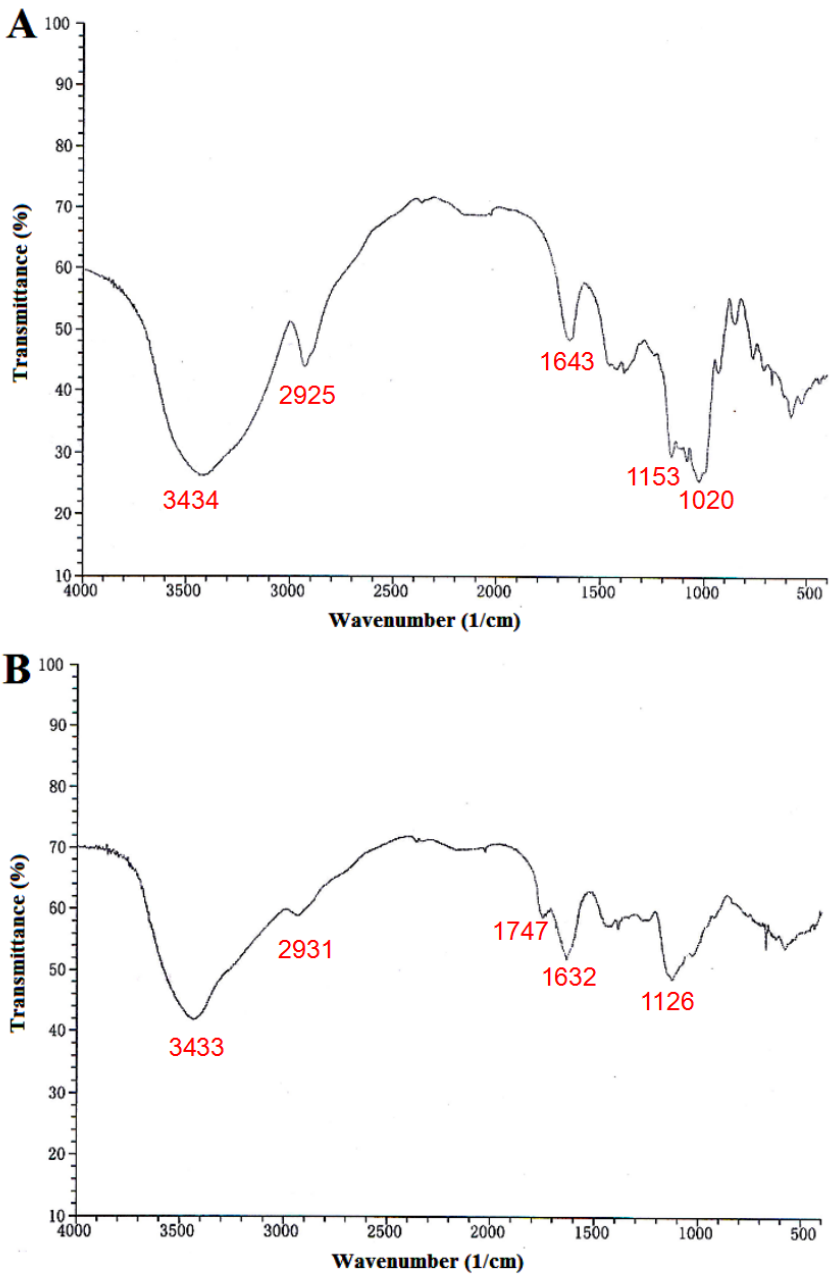

Figure 1. FT-IR spectrum of TPN-II (A) and TPA-II (B).

2. Results of NMR

NMR spectra were employed to further confirm the obtained structure data and provide more detailed structural information of TPN-II and TPA-II. As shown in Figure 2A,B, ^{1}H NMR spectrum crowded in a narrow region between 3 and 5.5 ppm. Typical characteristic signals of polysaccharides were shown in the spectrum, indicating the presence of sugar residues [6]. According to literature data, a set of wide and intense signals (δ 3.0 to δ4.0 ppm) were identified to be the structure of CH_2O and CHO groups of sugars [7]. Signals at δ 3.6 to δ4.1 ppm may be attributed to characteristic resonances of ring protons (H2–H5) [8]. The anomeric proton resonances of δ 4.5–5.5 ppm indicated the presence of α and β form glycoside bonds in TPN-II and TPA-II [9]. In addition, there was a proton signal at δ 4.7 ppm and no signal at 5.4 ppm, indicating that TPN-II was composed of α-glucopyranose [10].

^{13}C NMR spectrum shown in Figure 2C,D confirmed the results obtained by HPLC analysis and ^{1}H NMR. Signal at δ 55 to 86 ppm can be attributed to sugars C2–C5 according to the previous report [11]. Consistent with the results of ^{1}H NMR spectrum, the ^{13}C chemical shift of TPN-II was between 97 and 101 ppm, indicating that α form glucosyl linkage existed in its structure. In the ^{13}C NMR spectrum of TPA-II, signals appeared both in the ranges of δ 97–101 ppm and δ 103 - 106 ppm, proving two forms of glycoside bonds (α and β) in TPA-II [12,13]. No signal at low field at the range of 160 to 180 ppm was observed in the ^{13}C NMR spectrum of TPA-II, illustrating that it contains uronic acid (GluA and GalA) [14].

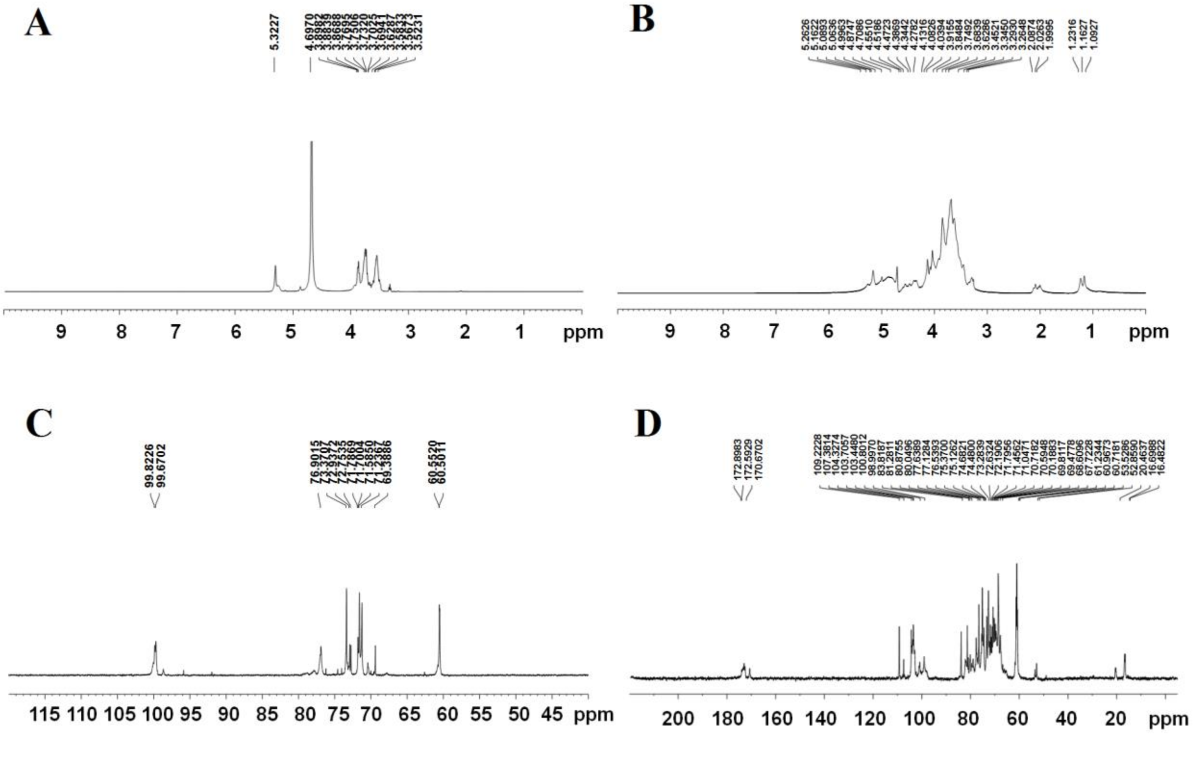

Figure 2. NMR spectrum of TPN-II and TPA-II. (A) ^{1}H NMR of TPN-II, (B) ^{1}H NMR of TPA-II, (C) ^{13}C NMR of TPN-II and (D) ^{13}C NMR of TPA-II.

References

1. Wang, Y.; Wei, X.L.; Wang, F.H.; Xu, J.J.; Tang, X.Z.; Li, N.Y. Structural characterization and antioxidant activity of polysaccharide from ginger. *Int. J. Biol. Macromol.* **2018,** 111, 862–869.
2. Ben Romdhane, M.; Haddar, A.; Ghazala, I.; Ben Jeddou, K.; Helbert, C.B.; Ellouz-Chaabouni, S. Optimization of polysaccharides extraction from watermelon rinds: Structure, functional and biological activities. *Food Chem.* **2017,** 216, 355–364.

3. Pawar, H.A.; Gavasane, A.J.; Choudhary, P.D. Extraction of polysaccharide from fruits of Cordia dichotoma G. Forst using acid precipitation method and its physicochemical characterization. *Int. J. Biol. Macromol.***2018,** *115*, 871–875.
4. Wang, J.L.; Wang, Y.X.; Xu, L.; Wu, Q.Q.; Wang, Q.; Kong, W.B.; Liang, J.Y.; Yao, J.; Zhang, J. Synthesis and structural features of phosphorylated Artemisia sphaerocephala polysaccharide. *Carbohyd. Polym.* **2018,** 181, 19–26.
5. He, P.F.; Zhang, A.Q.; Zhang, F.M.; Linhardt, R.J.; Sun, P.L. Structure and bioactivity of a polysaccharide containing uronic acid from Polyporus umbellatus sclerotia. *Carbohyd. Polym.* **2016,** 152, 222–230.
6. Li, J.W.; Fan, L.P.; Ding, S.D. Isolation, purification and structure of a new water-soluble polysaccharide from Zizyphus jujuba cv Jinsixiaozao. *Carbohyd. Polym.* **2011,** *83*, 477–482.
7. Ktari, N.; Feki, A.; Trabelsi, I.; Triki, M.; Maalej, H.; Ben Slima, S.; Nasri, M.; Ben Amara, I.; Ben Salah, R. Structure, functional and antioxidant properties in Tunisian beef sausage of a novel polysaccharide from Trigonella foenum-graecum seeds. *Int. J. Biol. Macromol.* **2017,** *98*, 169–181.
8. Kolsi, R.B.; Fakhfakh, J.; Krichen, F.; Jribi, I.; Chiarore, A.; Patti, F.P.; Blecker, C.; Allouche, N.; Belghith, H.; Belghith, K. Structural characterization and functional properties of antihypertensive Cymodocea nodosa sulfated polysaccharide. *Carbohyd. Polym.* **2016,** *151*, 511–522.
9. Fan, W.T.; Zhang, S.J.; Hao, P.; Zheng, P.M.; Liu, J.Z.; Zhao, X.N. Structure characterization of three polysaccharides and a comparative study of their immunomodulatory activities on chicken macrophage. *Carbohyd. Polym.* **2016,** *153*, 631–640.
10. Zhu, J.; Liu, W.; Yu, J.P.; Zou, S.; Wang, J.J.; Yao, W.B.; Gao, X.D. Characterization and hypoglycemic effect of a polysaccharide extracted from the fruit of Lycium barbarum L. *Carbohyd. Polym.* **2013,** *98*, 8–16.
11. Zhang, Q.; Xu, Y.; Lv, J.J.; Cheng, M.X.; Wu, Y.; Cao, K.; Zhang, X.F.; Mou, X.N.; Fan, Q. Structure characterization of two functional polysaccharides from Polygonum multiflorum and its immunomodulatory. *Int. J. Biol. Macromol.* **2018,** *113*, 195–204.
12. Shu, X.; Zhang, Y.F.; Jia, J.X.; Ren, X.J.; Wang, Y.F. Extraction, purification and properties of water-soluble polysaccharides from mushroom Lepista nuda. *Int. J. Biol. Macromol.* **2019,** *128*, 858–869.
13. Zhu, R.G.; Zhang, X.Y.; Wang, Y.; Zhang, L.J.; Zhao, J.; Chen, G.; Fan, J.G.; Jia, Y.F.; Yan, F.W.; Ning, C. Characterization of polysaccharide fractions from fruit of Actinidia arguta and assessment of their antioxidant and antiglycated activities. *Carbohyd. Polym.* **2019,** *210*, 73–84.
14. Meng, M.; Cheng, D.; Han, L.R.; Chen, Y.Y.; Wang, C.L. Isolation, purification, structural analysis and immunostimulatory activity of water-soluble polysaccharides from Grifola Frondosa fruiting body. *Carbohyd. Polym.* **2017,** *157*, 1134–1143.
